# Engineering CHO cell growth by stable manipulation of miRNA expression

**DOI:** 10.1186/1753-6561-5-S8-P22

**Published:** 2011-11-22

**Authors:** Noëlia Sanchez, Nga Lao, Clair Gallagher, Martin Clynes, Niall Barron

**Affiliations:** 1 National Institute for Cellular Biotechnology, Dublin City University, Dublin 9, Ireland

## Background

MiRNAs are small non-coding RNAs involved in many biological functions such as cell proliferation and apoptosis (1), cell cycle (2), homeostasis (3) and cell metabolism (4). They are highly conserved between species. They are capable of regulating hundreds of genes in a post-transcriptional manner by translation repression and/or mRNA degradation (5). These characteristics make miRNAs attractive tools for CHO cell engineering as multiple genes may be targeted simultaneously and possibly entire biological pathways can be manipulated. After temperature-shifting CHO cell culture from 37°C to 31°C, several miRNAs were found differentially regulated of which miR-7 was found to be down-regulated. Our laboratory has previously demonstrated that transient overexpression of miR-7 using mimics of mature endogenous miR-7, leads to a significant decrease in cell growth (6). On the other hand, transient inhibition using inhibitors of mature endogenous miR-7 provoked increased cell growth, though to a lesser extent. As this antisense technology is transient, another strategy was chosen to study the impact of stable miR-7 inhibition on CHO phenotypes. “Sponge” technology is an effective tool to stably sequester endogenous miRNAs (7) by introducing a decoy target reporter gene. This approach can be used to understand post-transcriptional regulation in CHO cells and to identify cellular pathways related to cell growth, cell viability and cell productivity - key phenotypes for cell bioprocess improvement.

## Materials and methods

Binding sites for either miR-7 (sponge miR-7) or a non-specific control sequence (sponge NC) were inserted into pCMV-deGFP (Professor.Sharp’s laboratory). SEAP-secreting CHO-K1 cells were transfected with 5µg of these constructs in a 6-well plate. Selection pressure was applied on day 1 after transfection by addition of hygromycin at 350µg/ml. Cells were transferred to a T-75 flask. From the mixed population, cells with high and medium GFP positivity were selected for single cell sorting using FACS flow cytometer in 96-well plates. Expanded clones were transferred to 24-well plates in adherent and suspension culture. GFP positivity and cell growth were monitored by Guava EasyCyte flow cytometer.

## Results

### Design of sponge NC/miR-7

Four tandemly repeated miR-7 binding sites were cloned downstream of a destabilised enhanced GFP to increase efficacy of endogenous miRNA sequestration. The negative control consisted of the same cassette with non-specific sequences to replace miR-7 binding sites. The deGFP, a modified enhanced GFP, consists of a fusion of the ornithine decarboxylase degradation domain from mouse (MODC) and the C-terminal of an enhanced variant of GFP (eGFP) conferring a shorter half-life than eGFP (2h). The change of deGFP fluorescence induced by the binding of endogenous miR-7 to the sponge can be correlated to the change in active miRNA levels. To avoid RNAi-type cleavage by Argonaute 2 and degradation, the sponges were designed with a bulge region (position 9-11).

### Impact of sponge miR-7 on CHO phenotypes in a mixed population

After transfection of the sponge miR-7 and sponge NC in SEAP-secreting CHO-K1 cells, GFP positivity and cell growth were monitored in both mixed populations and single clones. In mixed populations, sponge miR-7 transfected cells were found to have reduced GFP positivity compared to control pools (sponge miR-7: 34.9% and control 56.1%) and significantly improved cell density at day 3 (1.6x10^6^ cells/ml versus 1.16x10^6^ cells/ml respectively) and at day 4 of culture (3.4x10^6^ cells/ml and 2.9x10^6^ cells/ml, respectively).

### Impact of sponge miR-7 on CHO phenotypes in single clones

**Figure 1:**
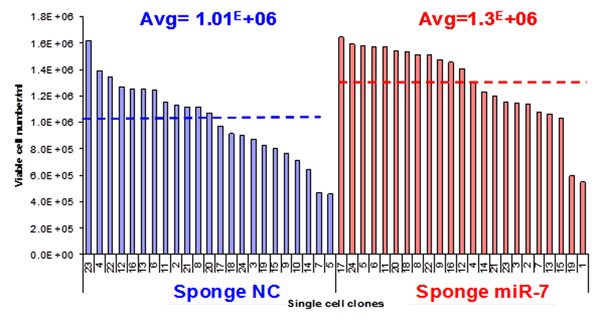
**CHO-K1 SEAP cell growth in sponge NC and sponge miR-7 expressing cell clones at day 3 of cell culture.***P*-value=0.0024 in student t-test.

In single clones, the average of GFP positivity was lower in sponge miR-7 expressing clones than in control clones (49.6% compared to 30.9%). The average cell density for 23 clones was significantly higher in sponge miR-7 expressing clones than in the control expressing clones (1.3x10^6^cells/ml and 1.01x10^6^cells/ml). Moreover, 12 clones from the sponge miR-7 expressing clones achieved more than 1.4x10^6^cells/ml whereas only one clone from the control expressing cells could reached this density. (Figure 1).

## Conclusions

In this study, we showed that miRNAs may be used as tools to improve CHO phenotypes, in this case cell density, and also as potential tools for understanding of CHO cellular pathway regulation. The application of miRNA expression manipulation in large-scale culture could be a novel technology to increase significantly the performance of biopharmaceutical production processes.
